# Rhodium-catalyzed homo-coupling reaction of aryl Grignard reagents and its application for the synthesis of an integrin inhibitor

**DOI:** 10.3762/bjoc.20.118

**Published:** 2024-06-12

**Authors:** Kazuyuki Sato, Satoki Teranishi, Atsushi Sakaue, Yukiko Karuo, Atsushi Tarui, Kentaro Kawai, Hiroyuki Takeda, Tatsuo Kinashi, Masaaki Omote

**Affiliations:** 1 Faculty of Pharmaceutical Sciences, Setsunan University, 45-1 Nagaotoge-cho, Hirakata, Osaka, 573-0101, Japan; 2 Kyowa Marina Hospital, 4-15-1 Nishinomiyahama, Nishinomiya, Hyogo 662-0934, Japan; 3 Division of Proteo-Drug-Discovery Sciences, Ehime University Proteo-Science Center; 4 Bunkyo-cho, Matsuyama, Ehime, 790-8577, Japan,; 5 Department of Molecular Genetics, Institute of Biomedical Science, Kansai Medical University, 2-5-1 Shin-machi, Hirakata, Osaka, 573-1010, Japan

**Keywords:** biphenyltetracarboxylic acid, homo-coupling, integrin inhibitor, rhodium catalyst, Ullmann-type reaction

## Abstract

A novel Rh-catalyzed one-pot homo-coupling reaction of aryl Grignard reagents was achieved. The reaction with bromobenzenes having an electron-donating group or a halogen substituent gave the corresponding homo-coupling products in good yields, although the reaction using heterocyclic or aliphatic bromides scarcely proceeded. A Rh(III)–bis(aryl) complex, which might be formed from RhCl(PPh_3_)_3_ and the aryl Grignard reagents, plays an important role in giving the homo-coupling products in this reaction. Furthermore, we applied the reaction to the synthesis of a novel inhibitor for integrins which is critical for several diseases.

## Introduction

**Scheme 1:**
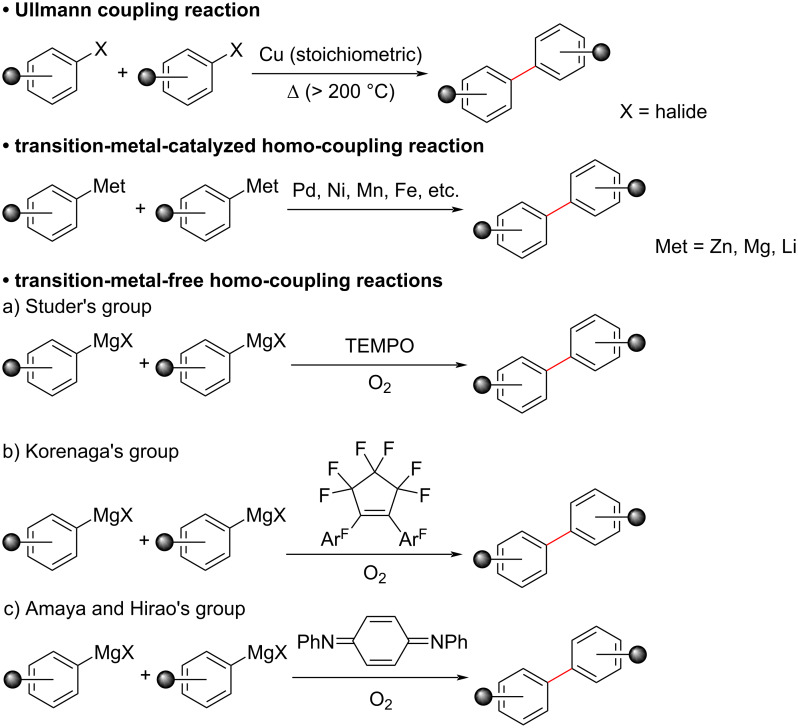
Ullmann and Ullmann-type homo-coupling reactions.

The Ullmann reaction is a coupling reaction of aryl halides using copper, traditionally using metallic copper-bronze alloy, and has been used as one of the methods for obtaining homo-coupling biaryl compounds (Scheme 1) [1–2]. Starting from these works, various modified Ullmann-type coupling reactions have been developed [3–4]. However, the reaction usually required high temperatures and the yield was not very high. Therefore, alternative procedures for homo-coupling reactions using other transition-metal catalysts such as palladium, nickel, manganese, and iron have been developed [5–13]. In recent years, transition-metal-free coupling reactions have also been developed for environmentally benign synthetic applications [14–18].

**Scheme 2:**
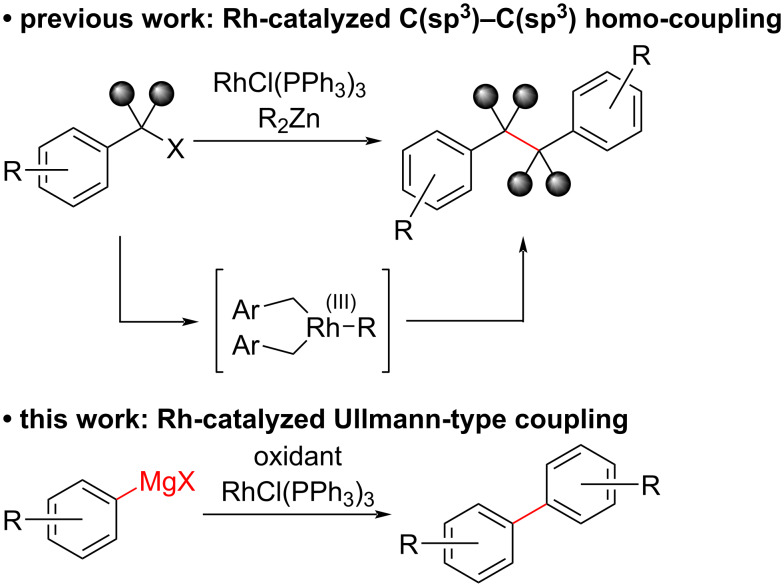
Rh-catalyzed homo-coupling reactions.

We have successfully achieved various reactions using a Rh complex easily derived from dialkylzinc (R_2_Zn) and RhCl(PPh_3_)_3_ [19–20]. Additionally, in the related study, we also reported a new Rh-catalyzed C(sp^3^)–C(sp^3^) homo-coupling reaction of benzyl halides, which involved a rhodium-bis(benzyl) complex (Scheme 2) [21]. Following these outcomes, as part of a research program aimed at a wide range of Rh-catalyzed C–C bond-formation reactions, in this paper, we report a Rh-catalyzed Ullmann-type homo-coupling reaction of aryl Grignard reagents.

## Results and Discussion

### Methodology development

**Scheme 3:**
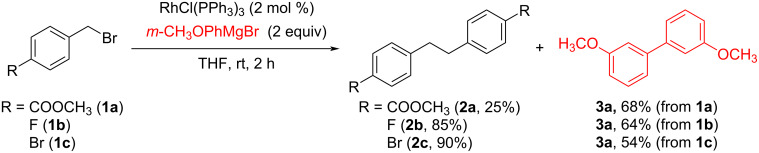
Rh-catalyzed homo-coupling reaction by using Grignard reagents.

In our work towards Rh-catalyzed homo-coupling reactions of benzyl halides, we observed that a similar rhodium–bis(benzyl) complex can also be formed from benzyl halide by using a Grignard reagent instead R_2_Zn in the presence of RhCl(PPh_3_)_3_ to subsequently give the desired dibenzyl product. For example, the reaction gave the desired dibenzyl product **2a** in 25% yield along with the corresponding biphenyl product **3a** in 68% yield, when methyl 4-(bromomethyl)benzoate (**1a**) was treated with 2.0 equiv of 3-methoxyphenylmagnesium bromide in the presence of 2 mol % of RhCl(PPh_3_)_3_ in THF as shown in Scheme 3. In addition, similar reactions using 4-fluorobenzyl bromide (**1b**) or 4-bromobenzyl bromide (**1c**) gave the desired dibenzyl products **2b** (85%) or **2c** (90%) along with **3a** in 64% or 54% yield, respectively.

Mechanistically, the benzyl halide works as an oxidizing agent, so various alkyl halides to replace the benzyl halides were investigated in this reaction (Table 1). Allyl bromide, propargyl bromide, and carbon tetrabromide did not work well as shown in entries 1, 2, and 6 in Table 1. On the other hand, other alkyl halides gave the product in moderate to good yields, especially 1,2-dibromoethane was the best oxidant. The reaction proceeded in good yield, even if 0.5 equiv of 1,2-dibromoethane were used in this reaction, as shown in Table 1, entries 7 and 8. However, it has been confirmed that this reaction did not proceed when no alkyl halide was added to the reaction mixture (Table 1, entry 10).

**Table 1:** Examination of various alkyl halides as an oxidant.

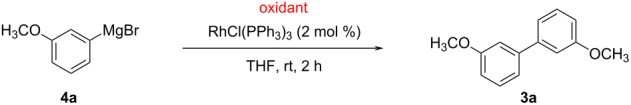							

entry	oxidant	equiv	yield (%)	entry	oxidant	equiv	yield (%)

1	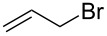	1	18	6	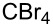	1	nd
2	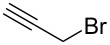	1	10	7	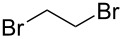	1	66
3	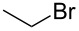	1	42	8	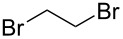	0.5	73
4	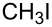	1	41	9	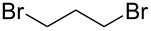	1	40
5	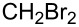	1	36	10	none	–	trace

Next, we investigated various Rh catalysts and solvents, and the results are summarized in Table 2. All Rh catalysts that were examined in this reaction gave the product in good yields, although in the absence of a Rh catalyst the reaction failed as shown in Table 2, entries 1–8. In addition, there was no need to prolong the reaction time (entry 3 in Table 2). Therefore, a Rh catalyst is essential in this reaction, and RhCl(PPh_3_)_3_ was chosen as the best catalyst because of its availability. In the examination of the reaction solvents, the use of THF led to the highest yield. It is interesting that the reaction proceeded without adding the oxidant (1,2-dibromoethane) when 1,2-dichloroethane was used as the solvent, although a slight decrease in yield was observed (Table 2, entry 14). Based on these results, we selected RhCl(PPh_3_)_3_ as the Rh catalyst and THF as the solvent as the best conditions (Table 2, entry 1).

**Table 2:** Examination of various Rh catalysts and solvents.

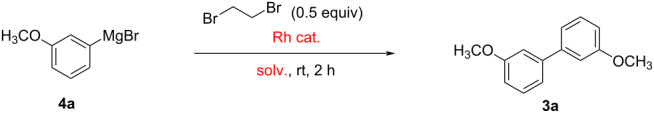							

entry	Rh cat (mol %)	solv.	yield (%)	entry	Rh cat.	solv.	yield (%)

1	RhCl(PPh_3_)_3_	THF	73	9	RhCl(PPh_3_)_3_	Et_2_O	48
2	RhCl(PPh_3_)_3_	THF	67	10	RhCl(PPh_3_)_3_	1,4-dioxane	48
3^a^	RhCl(PPh_3_)_3_	THF	67	11	RhCl(PPh_3_)_3_	toluene	65
4	RhCl(PPh_3_)_3_	THF	64	12	RhCl(PPh_3_)_3_	DCM	66
5	RhCl(CO)(PPh_3_)_2_	THF	61	13	RhCl(PPh_3_)_3_	DCE	67
6	[Rh(cod)Cl]_2_	THF	65	14^b^	RhCl(PPh_3_)_3_	DCE	58
7	Rh(CO)_2_(acac)	THF	65	15	RhCl(PPh_3_)_3_	DMF	trace
8	none	THF	trace				

^a^The reaction was carried out for 13 h. ^b^The reaction was carried out without oxidant.

**Scheme 4:**
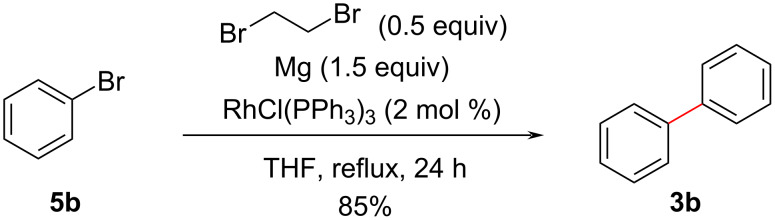
Rh-catalyzed one-pot Ullmann-type reaction with bromobenzene under optimized reaction conditions.

As shown above, the commercially available Grignard reagent **4a** gave the corresponding homo-coupled product **3a** in a short reaction time at room temperature. Subsequently, for the purpose of expanding the scope of substrates, we examined the in situ preparation of the Grignard reagent followed by the homo-coupling reaction. Various conditions were examined and biphenyl (**3b**) was obtained in 85% yield in a one-pot reaction, when bromobenzene (**5b**) was treated with 1.5 equiv of Mg (turnings, grade for Grignard reaction) under reflux conditions of THF for 24 h in the presence of RhCl(PPh_3_)_3_ (Scheme 4).

**Figure 1:**
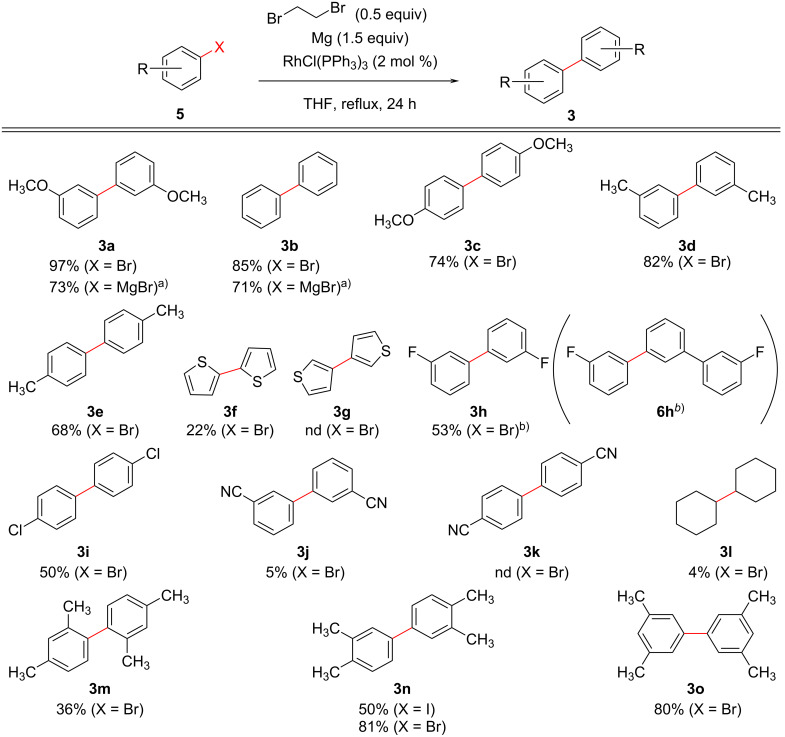
Scope and limitations for the Rh-catalyzed one-pot Ullmann-type reaction. Conditions: a) The reaction was carried out at rt for 1–3 h without Mg. b) The side product **6h** by S_N_Ar reaction onto **3h** was obtained in 8%.

Next, we investigated the Rh-catalyzed one-pot Ullmann-type reaction conditions by using various substrates, and the results are summarized in Figure 1. In the reaction with bromobenzenes bearing an electron-donating group such as bromotoluene and bromoanisole, the corresponding homo-coupling products **3a**, **3c**–**3e** were obtained in good yields. In addition, reactions with substrates having halogen substituents also gave the products (**3h** and **3i**) in moderate yields. It is interesting to note, that the reaction using 3-bromofluorobenzene (**5h**) gave a small amount of 3,3''-difluoro-1,1':3',1''-terphenyl (**6h**) as side product, that might derive from an S_N_Ar reaction of **3h** with the Grignard reagent of **5h**. Moreover, bromoxylenes **5m**–**o** also gave the corresponding products **3m**–**o**, respectively, although the position of substituents affected the yields. On the other hand, bromothiophenes **5f** and **5g**, bromobenzonitriles **5j** and **5k**, or cyclohexyl bromide (**5l**) were no suitable substrates for this reaction.

**Figure 2:**
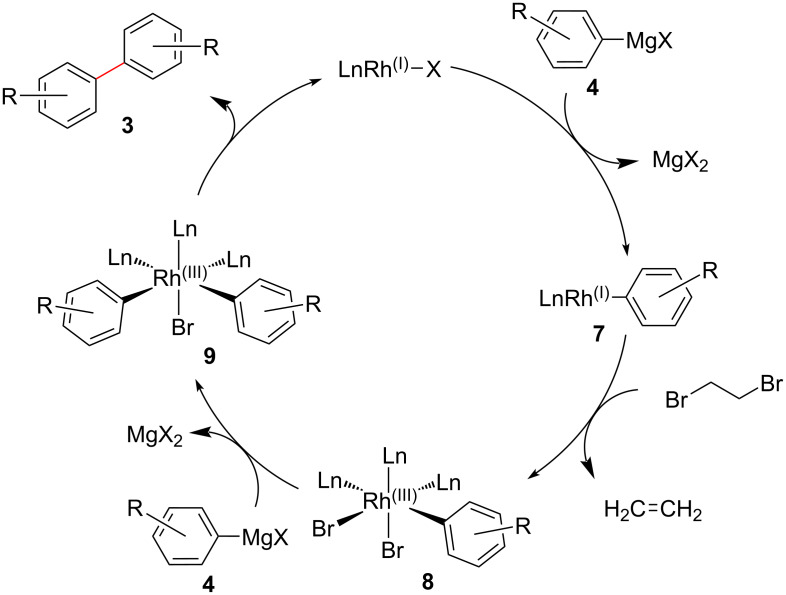
Tentative reaction mechanism.

Since this reaction did not proceed in the absence of a Rh catalyst, it was evident that a Rh complex is involved in this reaction, as already indicated by our previous results [21–22]. Consequently, we propose the reaction mechanism as shown in Figure 2. In the initial step, the Rh catalyst reacts with the Grignard reagent **4** to give the Rh(I)–aryl complex **7**. Oxidative addition of 1,2-dibromoethane onto complex **7** then generates Rh(III)–aryl complex **8** along with elimination of ethylene. Further transmetalation between the complex **8** and another Grignard reagent gives Rh(III)–bis(aryl) complex **9**. Finally, reductive elimination affords the desired homo-coupling product **3** and regenerates the Rh catalyst. We did not identify any cross-coupling products such as (2-bromoethyl)arenes or styrenes in this reaction. Unfortunately, we have not clarified the reason why a cross-coupling reaction did not proceed. At this stage, we speculate that the elimination rate of ethylene and reductive elimination rate of **3** might be fast in this reaction.

### Medicinal chemistry application

Integrins are transmembrane heterodimers, each consisting of α and β subunits, that mediate cell–cell and cell–matrix adhesion involved in normal and pathological processes. Bidirectional signaling through integrins regulate cell shape, motility, and cell cycle progression [23]. The integrin complexes are highly important for performing various cellular functions, such as cell proliferation, migration, and morphological changes [24–25]. Consequently, integrin inhibitors have received great attention as novel candidates for the treatment of several intractable diseases [26–28].

**Scheme 5:**
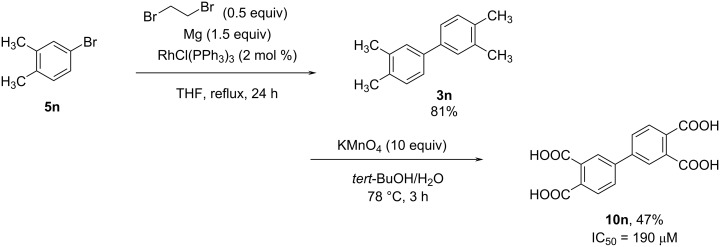
Synthesis of compound **10n** as a candidate for an integrin inhibitor.

In the process of developing novel inhibitors of integrin function, we identified a drug candidate (**10n**) through high throughput screening (HTS) that inhibits the integrin complex formation, which is an important step for integrin activation. The binding inhibitor **10n** was effective as IC_50_ of 190 μM in AlphaScreen system, and we thought that our homo-coupling reaction would be optimal for the synthesis of compound **10n**. Indeed, compound **5n** was successfully synthesized by the Rh-catalyzed homo-coupling reaction and subsequently oxidized by KMnO_4_ to give the desired product **10n** in 38% yield over two steps (Scheme 5) [29].

## Conclusion

In conclusion, we have developed a novel Rh-catalyzed one-pot Ullmann-type homo-coupling reaction of Grignard reagents. The use of commercially available Grignard reagents gave the corresponding homo-coupling products even when carried out at room temperature and short reaction time. However, since the preparation of Grignard reagents derived from haloarenes with Mg in situ requires heating for a long time, we decided to use heating conditions for the one-pot homo-coupling reaction. Most homo-coupling products were obtained in this reaction, although the product yield decreased when using substrates that are difficult to react with Mg to give the corresponding Grignard reagents. Furthermore, we successfully synthesized a candidate of integrin inhibitor by using the herein developed Rh-catalyzed Ullmann-type homo-coupling reaction as the key step. The integrin inhibitor was effective at an IC_50_ of 190 μM in an AlphaScreen system, and we believe that further QSAR studies of analogs of the inhibitor will lead to the discovery of novel potential therapeutics for the treatment of several intractable diseases.

## Supporting Information

File 1 General procedures and analytical data, including copies of ^1^H NMR and ^13^C NMR spectra.

## Data Availability

The data that supports the findings of this study is available from the corresponding author upon reasonable request.
