# Supplementary material for: Rhodium-catalyzed homo-coupling reaction of aryl Grignard reagents and its application for the synthesis of an integrin inhibitor
Source: Beilstein J Org Chem. 2024 Jun 12;20:1341–7. doi: 10.3762/bjoc.20.118 (PMC11181203; doi:10.3762/bjoc.20.118)

BEILSTEIN JOURNAL OF ORGANIC CHEMISTRY

Supporting Information

for

Rhodium-catalyzed homo-coupling reaction of aryl Grignard reagents and its application for the synthesis of an integrin inhibitor

Kazuyuki Sato, Satoki Teranishi, Atsushi Sakaue, Yukiko Karuo, Atsushi Tarui, Kentaro Kawai, Hiroyuki Takeda, Tatsuo Kinashi and Masaaki Omote

Beilstein J. Org. Chem. **2024,** *20,* 1341–1347. doi:10.3762/bjoc.20.118

General procedures and analytical data, including copies of ^{1}H NMR and ^{13}C NMR spectra

License and Terms: This is a supporting information file under the terms of the Creative Commons Attribution License (https://creativecommons.org/licenses/by/4.0). Please note that the reuse, redistribution and reproduction in particular requires that the author(s) and source are credited and that individual graphics may be subject to special legal provisions.
The license is subject to the *Beilstein Journal of Organic Chemistry* terms and conditions: (https://www.beilstein-journals.org/bjoc/terms)

Table of contents

General information ………………………………………………. S2
Materials …………………………………………….……………… S2
Experimental section
Typical procedure for the synthesis of **3a**
by using Grignard reagent ………………. S2
General procedure for Rh-catalyzed
one-pot Ullmann-type coupling reaction ………………. S3
Synthetic procedure for
[1,1'-biphenyl]-3,3',4,4'-tetracarboxylic acid ………S3
Inhibitory activity evaluation
against integrin complex formation ………S4
Spectroscopic data …………………………….…………….. S6–S9
References ……………………………………….…………… S10
NMR spectra ………………………………………………. S11–S21

General information

^{1}H NMR, ^{13}C NMR, and ^{19}F NMR spectra were recorded on JNM-GX400 spectrometers. Chemical shifts of ^{1}H NMR are reported in ppm from tetramethylsilane (TMS: 0 ppm) as an internal standard. Chemical shifts of ^{13}C NMR are reported in ppm from the solvent (DMSO-d_6: 39.520 ppm) as an internal standard. Chemical shifts of ^{19}F NMR are reported in ppm from trichlorofluoromethane ($CFCl_3$: 0 ppm) as an internal standard. All data are reported as follows: chemical shifts, relative integration value, multiplicity (s = singlet, d = doublet, t = triplet, q = quartet, br = broad, m = multiplet), coupling constants (Hz). Mass spectra were obtained on JEOL JMS-700T (EI or FAB) spectrometers.

Materials

Tetrahydrofuran (THF) was distilled over benzophenone ketyl sodium just before use. All commercially available reagents were used without further purification. All experiments were carried out under argon atmosphere in flame-dried glassware using standard inert techniques for introducing reagents and solvents unless otherwise noted.

Experimental section

Typical procedure for the synthesis of 3a by using Grignard reagent.

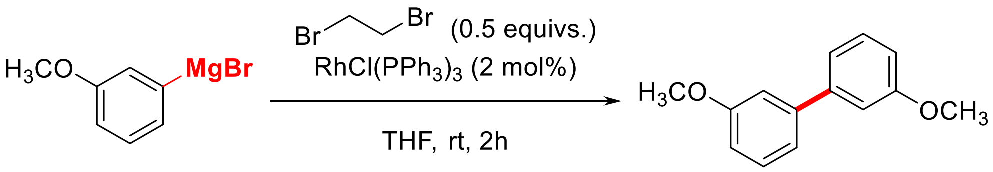

4a **3a**

To a solution of $RhCl(PPh_3)_3$ (2 mol %) and 1,2-dibromoethane (1.0 mmol) in THF (10 mL) was added 1.0 M 3-methoxyphenylmagnesium bromide in THF (2 mL, 2.0 mmol) at ambient temperature, and the mixture was stirred for 2 h at the same temperature. The resulting mixture was quenched with 10% HCl, and extracted with AcOEt. The AcOEt layer was washed with sat NaCl and dried over $MgSO_4$. The solvent was removed in vacuo and the residue was purified by column chromatography (SiO_2) to give 3,3'-dimethoxy-1,1'-biphenyl (**3a**) in 73% yield.

General procedure for Rh-catalyzed one-pot Ullmann-type coupling reaction

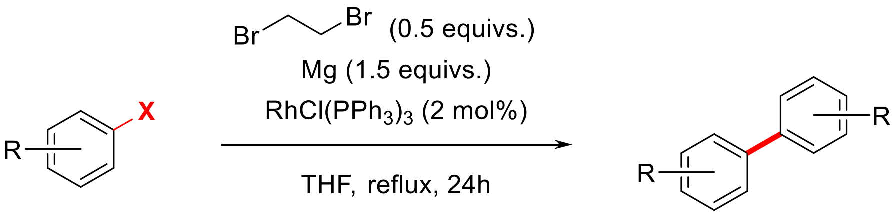

5 **3**

To a suspension of $RhCl(PPh_3)_3$ (2 mol %) and Mg (turnings, grade for Grignard reaction; 3 mmol) in THF (10 mL) was added the corresponding aryl halide **5** (2.0 mmol) and 1,2-dibromoethane (1.0 mmol), and the resulting mixture was refluxed for 24 h. The reaction was quenched with 10% HCl, and extracted with AcOEt. The AcOEt layer was washed with sat. NaCl and dried over $MgSO_4$. The solvent was removed in vacuo and the residue was purified by column chromatography (SiO_2) to give the product **3**.

Synthetic procedure for [1,1'-biphenyl]-3,3',4,4'-tetracarboxylic acid[1]

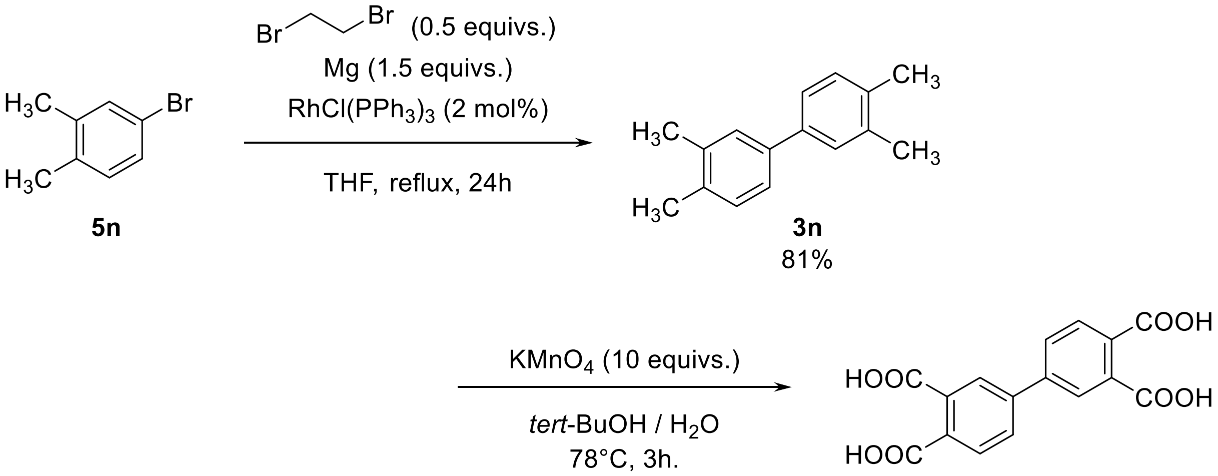

10n, 47%

According to the above procedure, compound **3n** was synthesized from 4-bromo-*o*-xylene (**5n**; 2.0 mmol). The reaction mixture was purified by column chromatography (SiO_2, hexane) to give **3n** (171 mg, 81%) as a colorless solid.

A solution of compound **3n** (1 mmol) in *tert*-butanol (1.25 mL) and water (1.25 mL) was heated at 78 °C, then $KMnO_4$ (10 mmol) was added directly to the mixture in small portions (over 15 min). After 3 h, to the reaction mixture cooled to ambient temperature was added sat. $Na_2S_2O_3$ and stirred overnight. The resulting mixture was filtered through Celite, the pH adjusted to 1 by the addition of conc. HCl to the mixture, and the mixture was stirred for 18 h. On cooling, the precipitate was collected and rinsed with water and AcOEt to give product **10n** (155 mg, 47%).

Inhibitory activity evaluation against integrin complex formation

To evaluate the integrin complex formation, we established the evaluation system using AlphaScreen technology.[2]

1. Construction of tagged recombinant cDNA plasmids

Genes encoding human integrin b2 cytoplasmic tail and talin FERM domain were amplified by PCR using human cDNA from Mammalian Gene Collection as templates[3]. Overlapping sequences were added at the 5′ and 3′ ends for seamless cloning. Amplified integrin β2 cytoplasmic tail and talin-1 FERM domain were subcloned into the pEU-E01-GW-FLAG-GST and pEU-E01-GW-His-bls vectors, respectively, using Gibson Assembly seamless cloning. Template DNA fragments for in vitro transcription were PCR-amplified using the SPu-2 primer (5′-CAGTAAGCCAGATGCTACAC-3′), AODA2306 primer (5′-AGCGTCAGACCCCGTAGAAA-3′), and with the pEU plasmids diluted by TE buffer.

2. Preparation of recombinant proteins using a wheat germ cell-free synthesis system

The recombinant FLAG-GST tagged human integrin β2 cytoplasmic tail and His-biotin tagged talin-1 FERM domains were synthesized using a wheat germ cell-free synthesis system[4]. Transcription and translation reactions were conducted using a WEPRO7240 Expression Kit (CellFree Sciences, Matsuyama, Japan). The transcription reaction mixture was prepared by mixing 2.6 μL of transcription buffer LM, 1.3 μL of NTP mixture (25 mM each), 0.26 μL of RNase inhibitor, 0.52 μL of SP6 polymerase, and 2.6 μL of PCR product. The transcription reaction was incubated at 37 °C for 18 h. Twenty-five microliters of the translation mixture containing 12.5 μL of mRNA, 8 μL of WEPRO 7240 wheat germ extract, 1 μL of creatine kinase (2 mg/mL, Roche Diagnostics, Basel, Switzerland), and 0.55 μL of RNase inhibitor were prepared and overlaid with 125 μL of translation buffer (SUB-AMIX SGC) in a 96-well plate. The biotin ligation site (bls) was biotinylated enzymatically by adding BirA biotin ligase and biotin (Sigma-Aldrich, St. Louis, MO, USA) to the translation mixture[5]. The plate containing the translation reaction was incubated at 15 °C for 24 h. After incubation, the reaction mixtures were mixed well, divided into small portions, and frozen in liquid nitrogen. The recombinant protein samples were stored at −80 °C until use.

3. Amplified luminescence proximity homogeneous assay

All ALPHA reactions were conducted in an 1/2 area OptiPlate-96 microplate (PerkinElmer, Waltham, MA, USA). All proteins and reagents were diluted in reaction buffer (100 mM Tris-HCl (pH 8.0), 0.01% Tween 20, and 1 mg/mL bovine serum albumin). The reaction total volume is 40 μL, and first, 10 μL of the solution containing 20 nL of a biotin-tagged domain in reaction buffer was dispensed into the reaction plate. Next, 10 μL of objective concentration of compounds was added into the well. Then, 20 nL of FLAG-tagged protein was transferred to

the reaction plate. After gentle mixing, 10 µL of detection mixture containing 20 nL of an anti-DYKDDDDK tag monoclonal antibody, 50 nL of streptavidin-conjugated AlphaScreen donor beads, and 50 nL of protein A-conjugated AlphaScreen acceptor beads in reaction buffer were added to each well of the reaction plate. After incubation at 26 °C for 1 h, the ALPHA chemiluminescence signal was detected by an EnSpire Plate Reader (Revvity). The signal data were analyzed using GraphPad Prism 8.

Spectroscopic data

3,3'-Dimethoxy-1,1'-biphenyl (3a)[6]

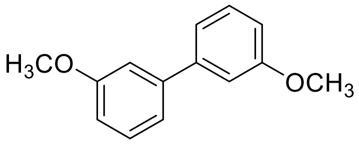

^{1}H NMR (400 MHz, $CDCl_3$) δ: 3.86 (6H, s), 6.89–6.91 (2H, m), 7.11–7.12 (2H, m), 7.16–7.19 (2H, m), 7.33–7.37 (2H, m); MS *m/z*: 214 (M^+); HRMS Calcd for $C_{14}H_{14}O_2$: 214.0994 (M^+), Found: 214.0998.

1,1'-Biphenyl (3b)[6-9]

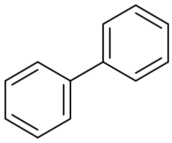

^{1}H NMR (400 MHz, $CDCl_3$) δ: 7.32–7.37 (2H, m), 7.42–7.46 (4H, m), 7.58–7.61 (4H, m); MS *m/z*: 154 (M^+); HRMS Calcd for $C_{12}H_{10}$: 154.0783 (M^+), Found: 154.0786.

4,4'-Dimethoxy-1,1'-biphenyl (3c)[6-9]

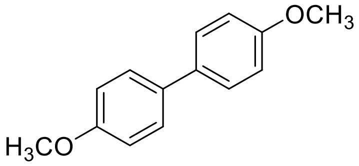

^{1}H NMR (400 MHz, $CDCl_3$) δ: 3.84 (6H, s), 6.94–6.97 (4H, m), 7.46–7.49 (4H, m); MS *m/z*: 214 (M^+); HRMS Calcd for $C_{14}H_{14}O_2$: 214.0994 (M^+), Found: 214.0995.

3,3'-Dimethyl-1,1'-biphenyl (3d)[6]

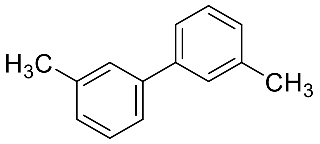

^{1}H NMR (400 MHz, $CDCl_3$) δ: 2.42 (6H, s), 7.14–7.16 (2H, m), 7.30–7.34 (2H, m), 7.37–7.40 (4H, m); MS *m/z*: 182 (M^+); HRMS Calcd for $C_{14}H_{14}$: 182.1096 (M^+), Found: 182.1098.

4,4'-Dimethyl-1,1'-biphenyl (3e)[6,7,9]

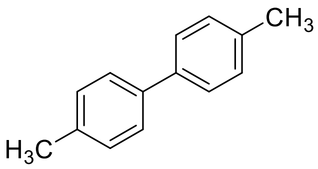

^{1}H NMR (400 MHz, $CDCl_3$) δ: 2.38 (6H, s), 7.22–7.25 (4H, m), 7.46–7.49 (4H, m); MS *m/z*: 182 (M^+); HRMS Calcd for $C_{14}H_{14}$: 182.1096 (M^+), Found: 182.1097.

2,2'-Bithiophene (3f)[7,9]

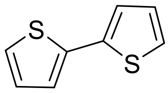

^{1}H NMR (400 MHz, $CDCl_3$) δ: 6.99–7.01 (2H, m), 7.16–7.20 (4H, m); MS *m/z*: 166 (M^+); HRMS Calcd for $C_8H_6S_2$: 165.9911 (M^+), Found: 165.9902.

3,3'-Difluoro-1,1'-biphenyl (3h)[8]

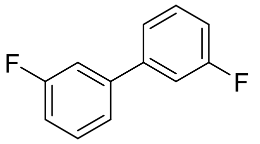

^{1}H NMR (400 MHz, $CDCl_3$) δ: 7.01–7.06 (2H, m), 7.21–7.25 (2H, m), 7.30–7.39 (4H, m); ^{19}F NMR (376 MHz, $CDCl_3$) δ: -112.5 (2F, m); MS *m/z*: 190 (M^+); HRMS Calcd for $C_{12}H_8F_2$: 190.0594 (M^+), Found: 190.0599.

4,4'-Dichloro-1,1'-biphenyl (3i)[6]

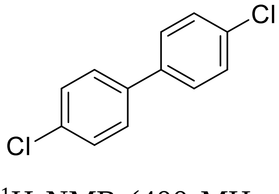

^{1}H NMR (400 MHz, $CDCl_3$) δ: 7.40–7.42 (4H, m), 7.46–7.49 (4H, m); MS *m/z*: 222 (M^+); HRMS Calcd for $C_{12}H_8Cl_2$: 222.0003 (M^+), Found: 221.9999.

[1,1'-Biphenyl]-3,3'-dicarbonitrile (3j)[9]

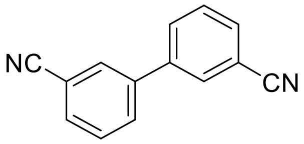

^{1}H NMR (400 MHz, $CDCl_3$) δ: 7.60–7.63 (2H, m), 7.71–7.73 (2H, m), 7.79–7.81 (2H, m), 7.85 (2H, m); MS *m/z*: 204 (M^+); HRMS Calcd for $C_{14}H_8N_2$: 204.0687 (M^+), Found: 204.0690.

1,1'-Bi(cyclohexane) (3l)[7]

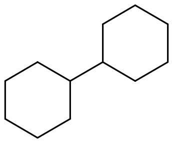

^{1}H NMR (400 MHz, $CDCl_3$) δ: 0.90–1.26 (12H, m), 1.62–1.73 (10H, m); MS *m/z*: 166 (M^+); HRMS Calcd for $C_{12}H_{22}$: 166.1722 (M^+), Found: 166.1720.

2,2',4,4'-Tetramethyl-1,1'-biphenyl (3m)[6]

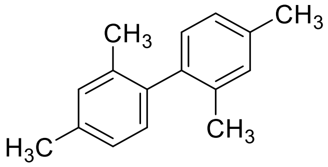

^{1}H NMR (400 MHz, $CDCl_3$) δ: 2.03 (6H, s), 2.36 (6H, s), 6.97–7.04 (4H, m), 7.08 (2H, m); MS *m/z*: 210 (M^+); HRMS Calcd for $C_{16}H_{18}$: 210.1409 (M^+), Found: 210.1407.

3,3',4,4'-Tetramethyl-1,1'-biphenyl (3n)

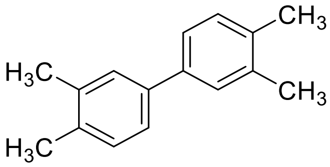

^{1}H NMR (400 MHz, $CDCl_3$) δ: 2.29 (6H, s), 2.32 (6H, s), 7.17–7.19 (2H, m), 7.30–7.32 (2H, m), 7.35–7.36 (2H, m); ^{13}C NMR (100 MHz, $CDCl_3$) δ: 19.41, 19.93, 124.3, 128.3, 130.0, 135.3, 136.8, 138.9; MS *m/z*: 210 (M^+); HRMS Calcd for $C_{16}H_{18}$: 210.1409 (M^+), Found: 210.1409.

3,3',5,5'-Tetramethyl-1,1'-biphenyl (3o)

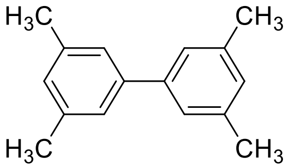

^{1}H NMR (400 MHz, $CDCl_3$) δ: 2.37 (12H, s), 6.97–6.98 (2H, m), 7.19 (4H, m); ^{13}C NMR (100 MHz, $CDCl_3$) δ: 21.40, 125.1, 128.7, 138.1, 141.5; MS *m/z*: 210 (M^+); HRMS Calcd for $C_{16}H_{18}$: 210.1409 (M^+), Found: 210.1409.

3,3''-Difluoro-1,1':3',1''-terphenyl (6h)

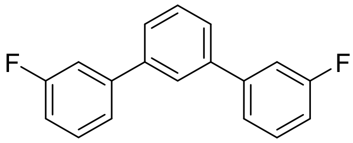

^{1}H NMR (400 MHz, $CDCl_3$) δ: 7.04–7.09 (2H, m), 7.31–7.35 (2H, m), 7.38–7.45 (4H, m), 7.50–7.58 (3H, m), 7.75 (1H, m); ^{13}C NMR (100 MHz, $CDCl_3$) δ: 114.1 (d, J = 19.3 Hz), 114.4 (d, J = 18.5 Hz), 122.9 (d, J = 2.6 Hz), 126.0, 126.6, 129.4, 130.3 (d, J = 8.4 Hz) 140.7 (d, J = 1.9 Hz), 143.2 (d, J = 7.6 Hz), 163.2 (d, J = 245.9 Hz); ^{19}F NMR (376 MHz, $CDCl_3$) δ: -112.8 (2F, m); MS *m/z*: 266 (M^+).

[1,1'-Biphenyl]-3,3',4,4'-tetracarboxylic acid (10n)

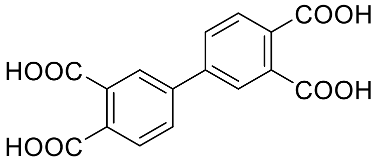

^{1}H NMR (400 MHz, DMSO-d_6) δ: 7.81–7.83 (2H, m), 7.96–8.00 (4H, m); ^{13}C NMR (100 MHz, DMSO-d_6) δ: 126.7, 129.1, 129.6, 132.1, 134.2, 140.7, 168.3, 168.6; MS *m/z*: 294 ($M^+ - 2H_2O$); HRMS Calcd for $C_{16}H_6O_6$: 294.0164 ($M^+ - 2H_2O$), Found: 294.0163; MS (FAB^+) *m/z*: 331 (M^+ + H); HRMS Calcd for $C_{16}H_{11}O_8$: 331.0454 (M^+ + H), Found: 331.0457.

References:

1. Hayashi, T. JP2009079008A, 2009-04-16.
2. Zhou, W.; Kaneko, N.; Nakagita, T.; Takeda, H.; Masumoto, J. *Cell Death Differ.* **2021**, *28*, 2991–3008.
3. Mammalian Gene Collection Program Team *Proc. Natl. Acad. Sci.* **2002**, *99*, 16899–16903.
4. Takai, K.; Sawasaki, T.; Endo, Y. *Nat. Protoc.* **2010**, *5*, 227–238.
5. Sawasaki, T.; Kamura, N.; Matsunaga, S.; Saeki, M.; Tsuchimochi, M.; Morishita, R.; Endo, Y. *FEBS Lett.* **2008**, *582*, 221–228.
6. Zhang, S.; Kolluru, L.; K. Vedula, S.; Whippie, D.; Jin, J. *Tetrahedron Lett.* **2017**, *58*, 3594–3597.
7. Xu, X.; Cheng, D.; Pei, W. *J. Org. Chem.* **2006**, *71*, 6637–6639.
8. Wang, Z.-Y.; Ma, Q.-N.; Li, R.-H.; Shao, L.-X. *Org. Biomol. Chem.* **2013**, *11*, 7899–7906.
9. Cheng, G.; Luo, M. *Eur. J. Org. Chem.* **2011**, *2011*, 2519–2523.

NMR spectra

^{1}H NMR of 3,3'-dimethoxy-1,1'-biphenyl (**3a**)

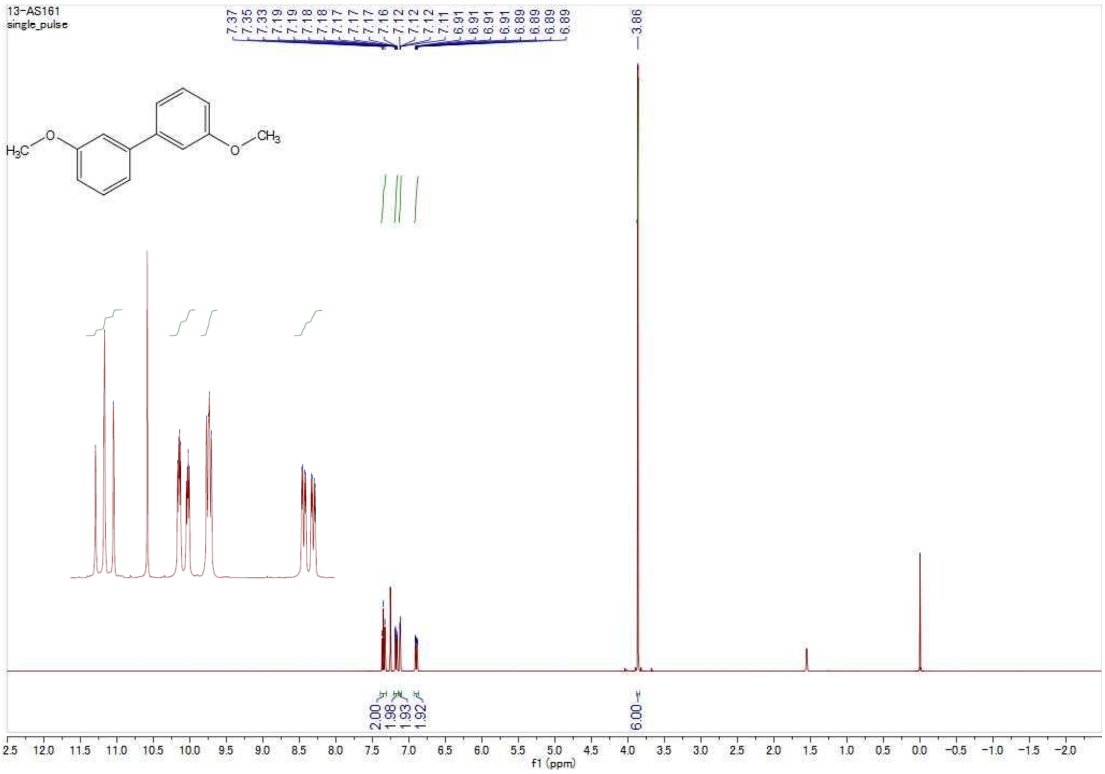

^{1}H NMR of 1,1'-biphenyl (**3b**)

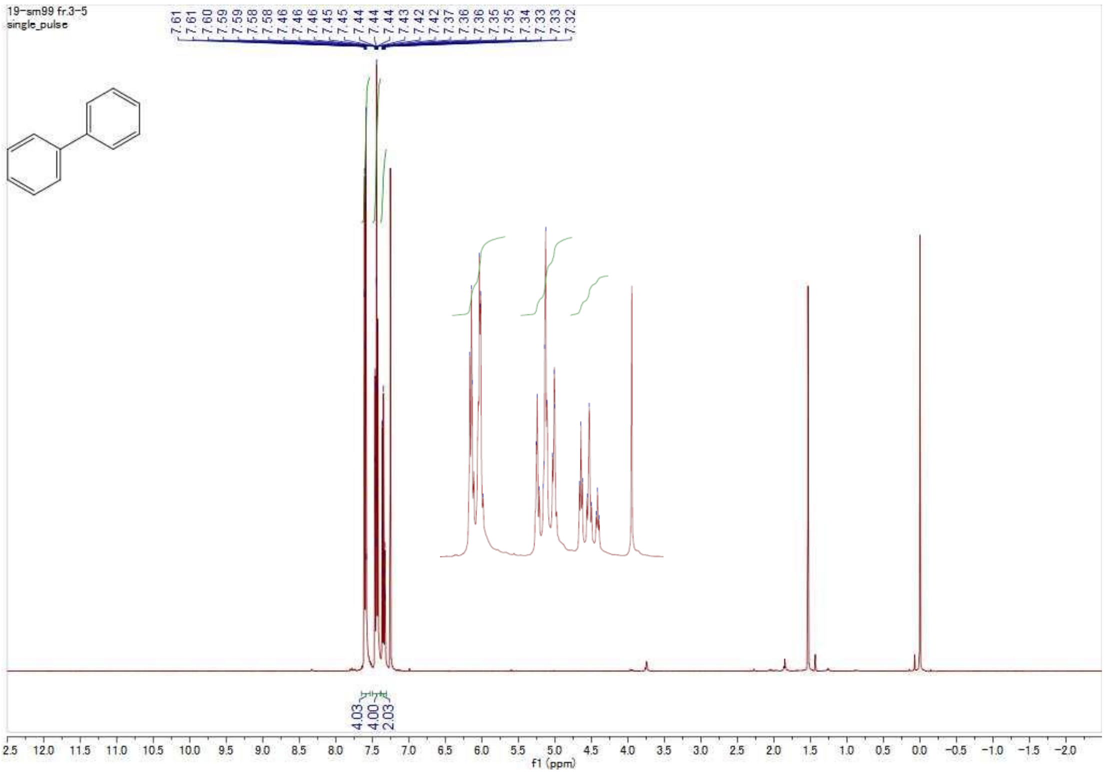

^{1}H NMR of 4,4'-dimethoxy-1,1'-biphenyl (**3c**)

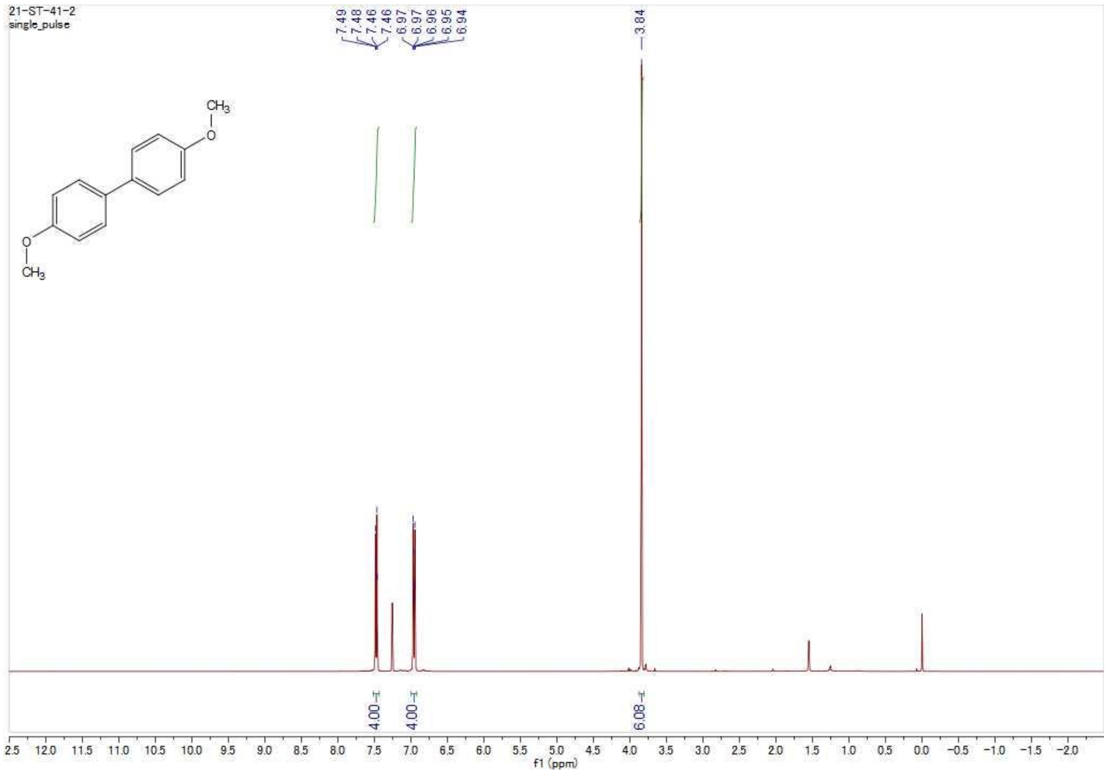

^{1}H NMR of 3,3'-dimethyl-1,1'-biphenyl (**3d**)

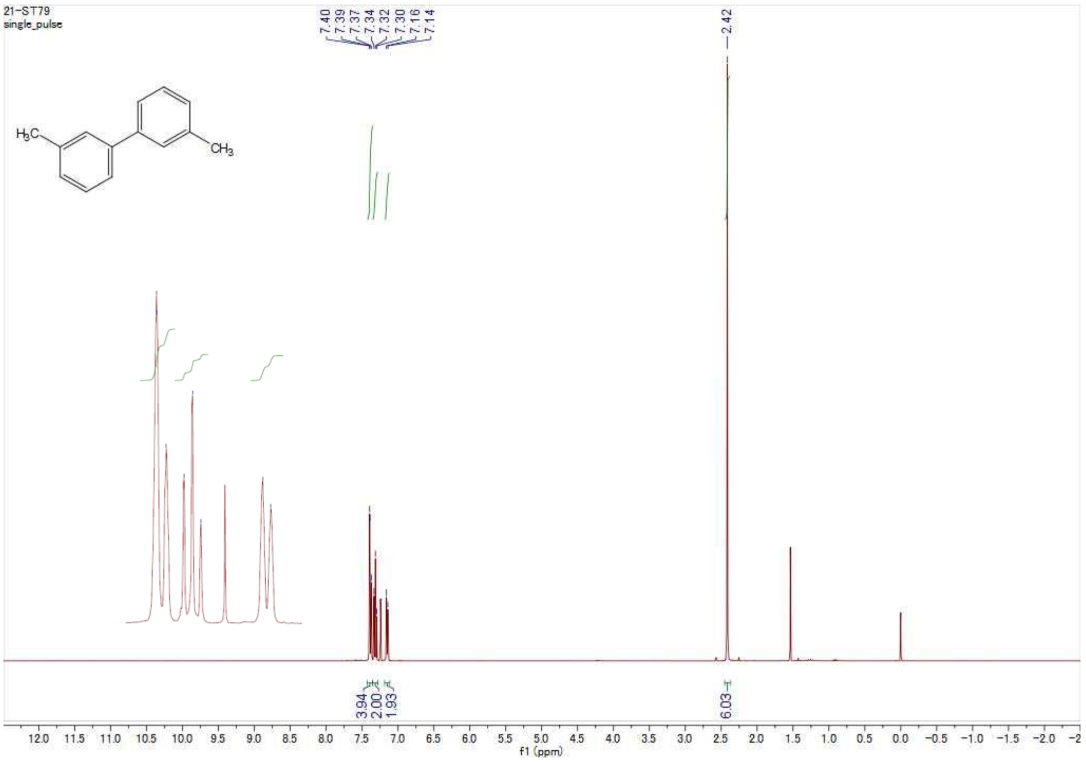

^{1}H NMR of 4,4'-dimethyl-1,1'-biphenyl (**3e**)

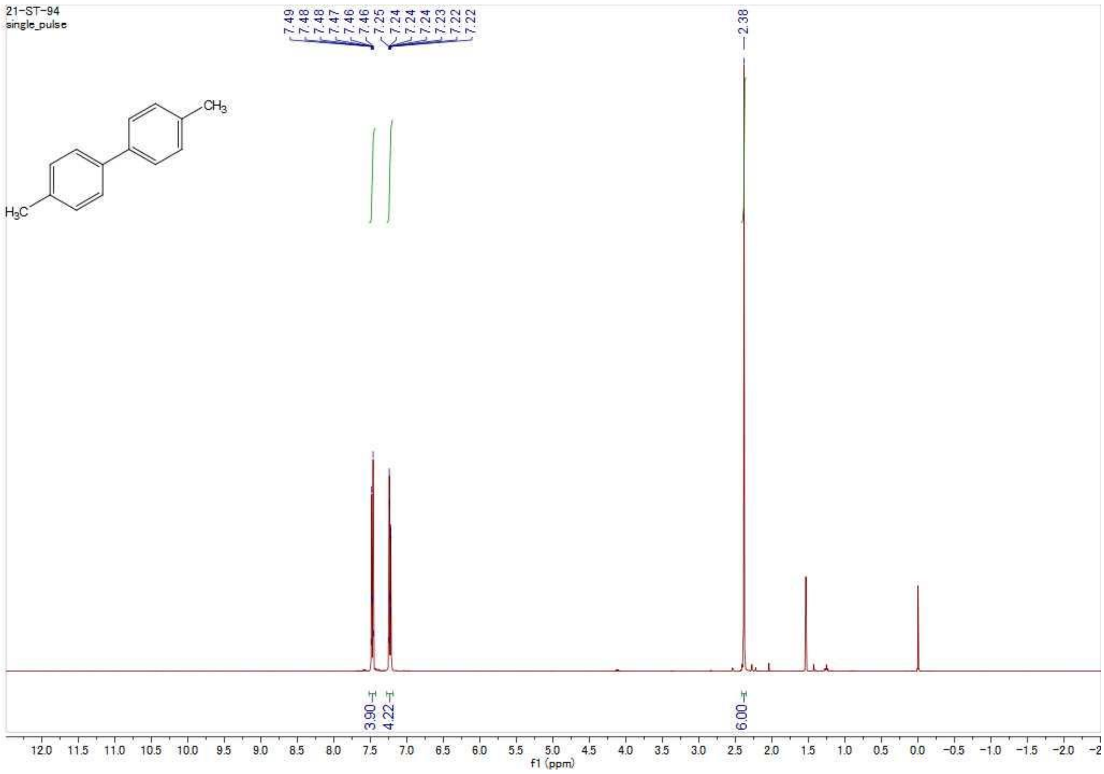

^{1}H NMR of 2,2'-bithiophene (**3f**)

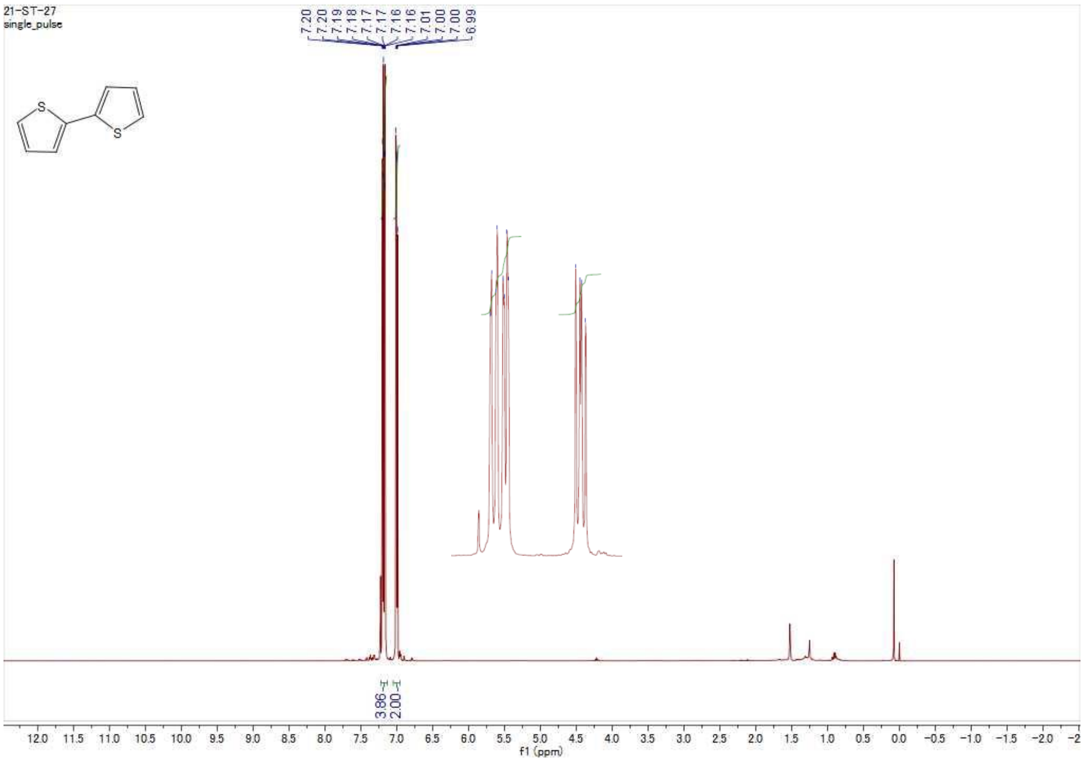

^{1}H NMR of 3,3'-difluoro-1,1'-biphenyl (**3h**)

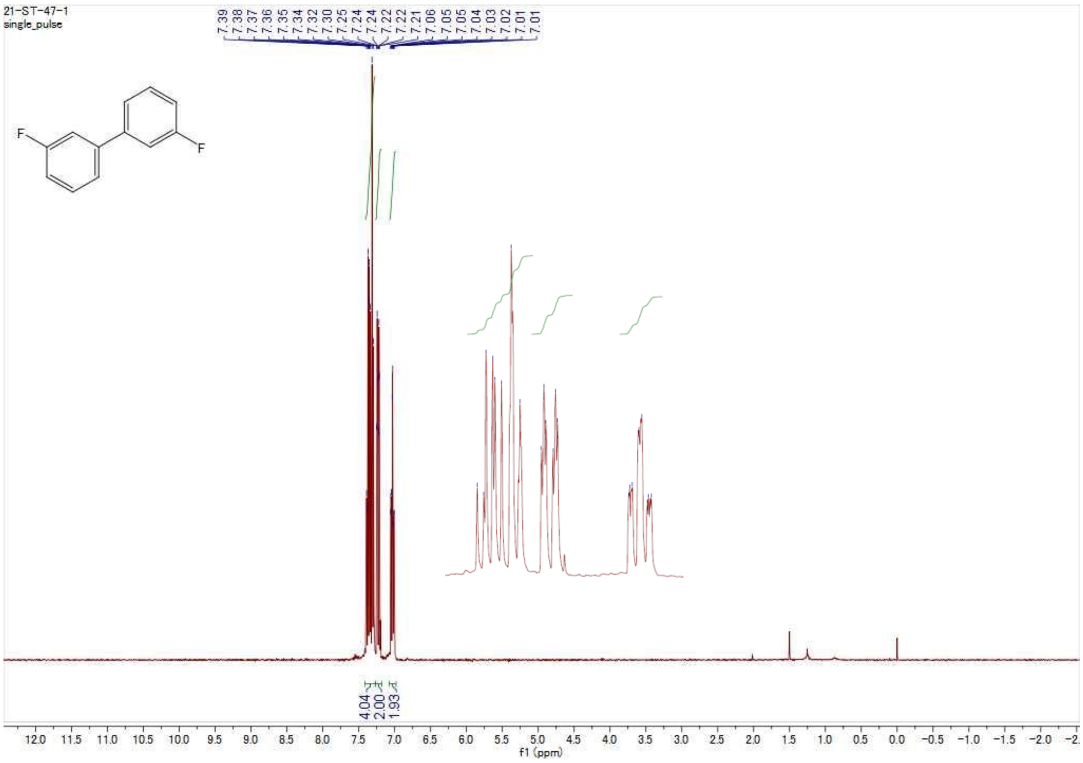

^{19}F NMR of 3,3'-difluoro-1,1'-biphenyl (**3h**)

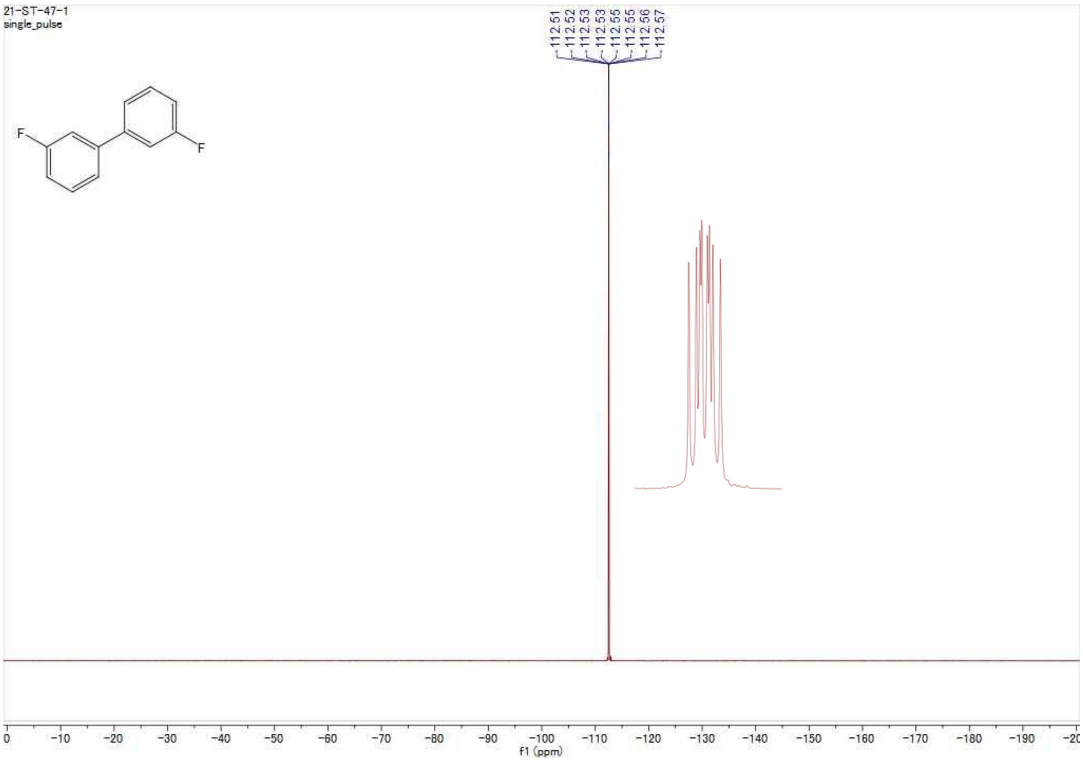

^{1}H NMR of 4,4'-dichloro-1,1'-biphenyl (**3i**)

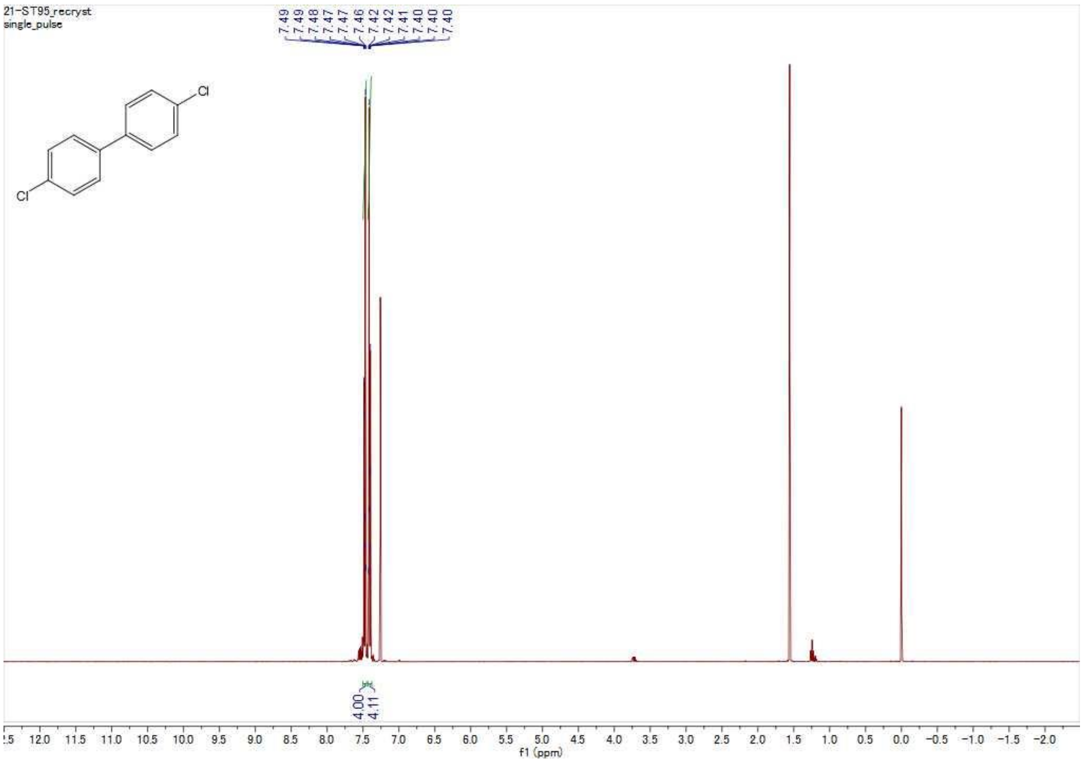

^{1}H NMR of [1,1'-biphenyl]-3,3'-dicarbonitrile (**3j**)

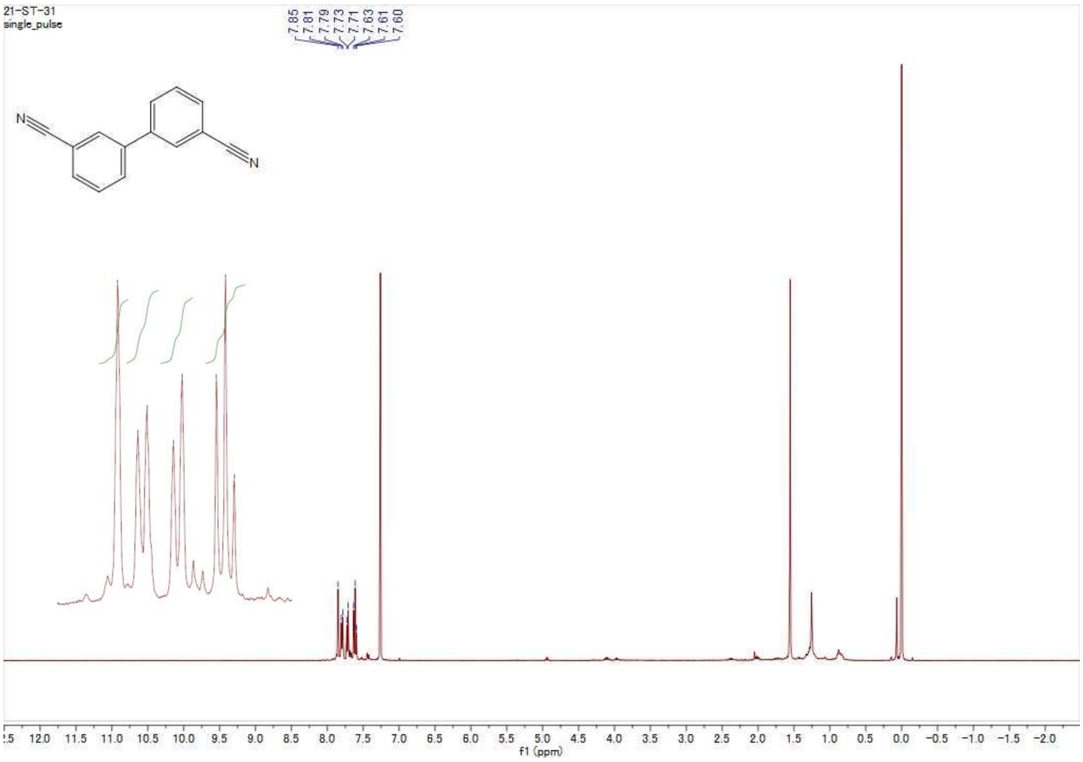

^{1}H NMR of 1,1'-bi(cyclohexane) (**3l**)

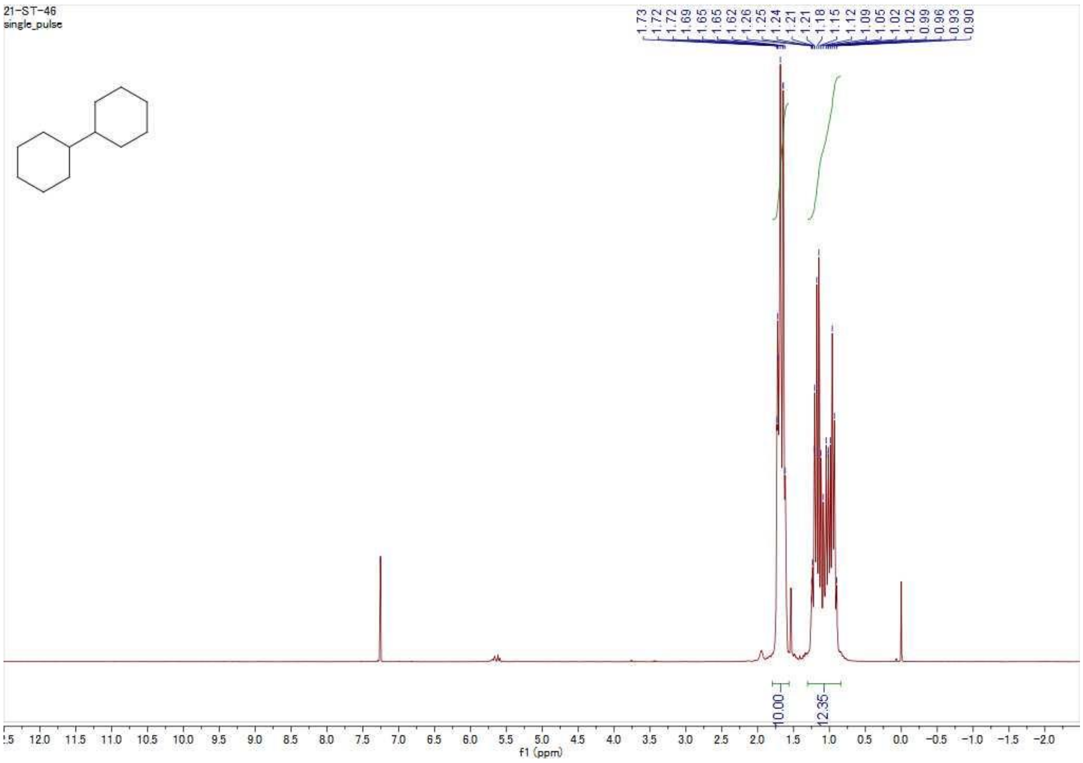

^{1}H NMR of 2,2',4,4'-tetramethyl-1,1'-biphenyl (**3m**)

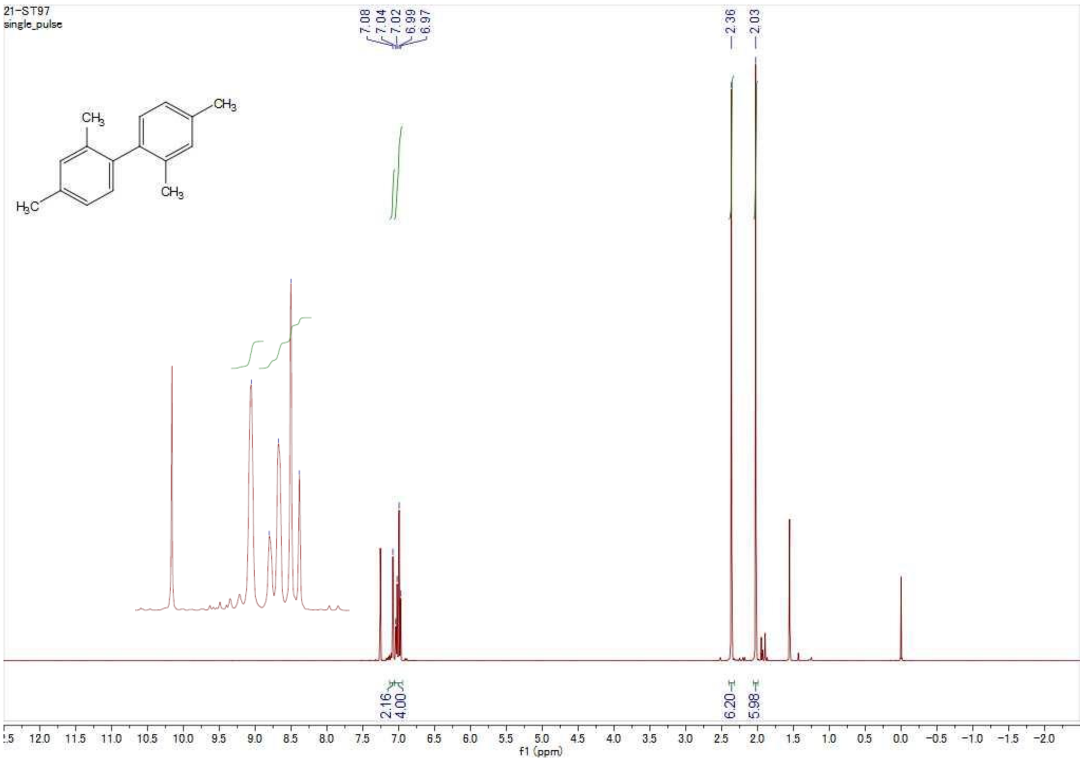

^{1}H NMR of 3,3',4,4'-tetramethyl-1,1'-biphenyl (**3n**)

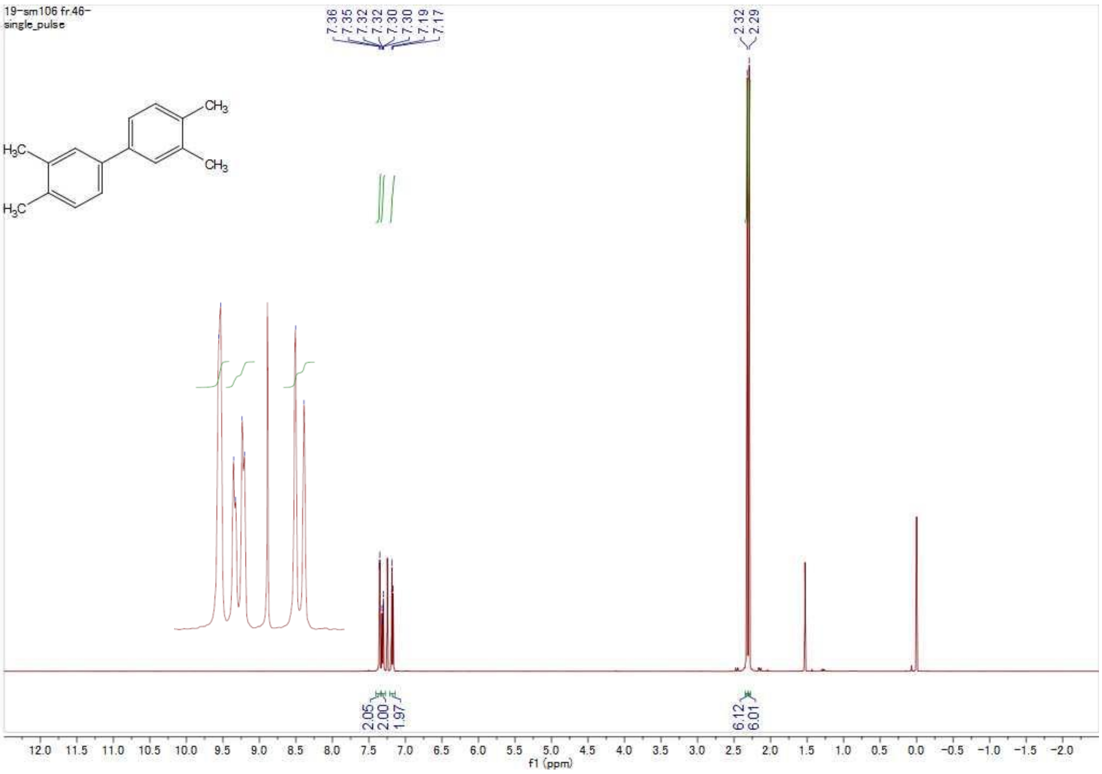

^{13}C NMR of 3,3',4,4'-tetramethyl-1,1'-biphenyl (**3n**)

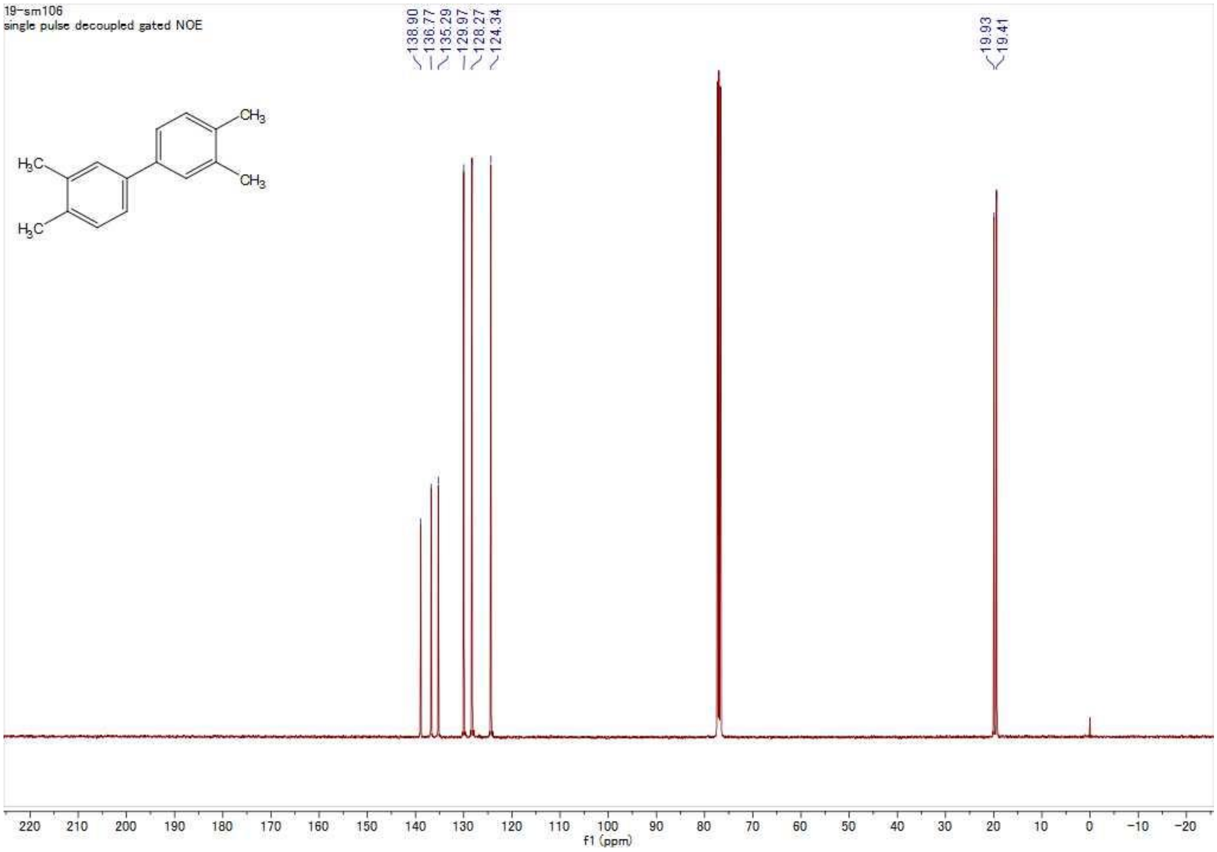

^{1}H NMR of 3,3',5,5'-tetramethyl-1,1'-biphenyl (**3o**)

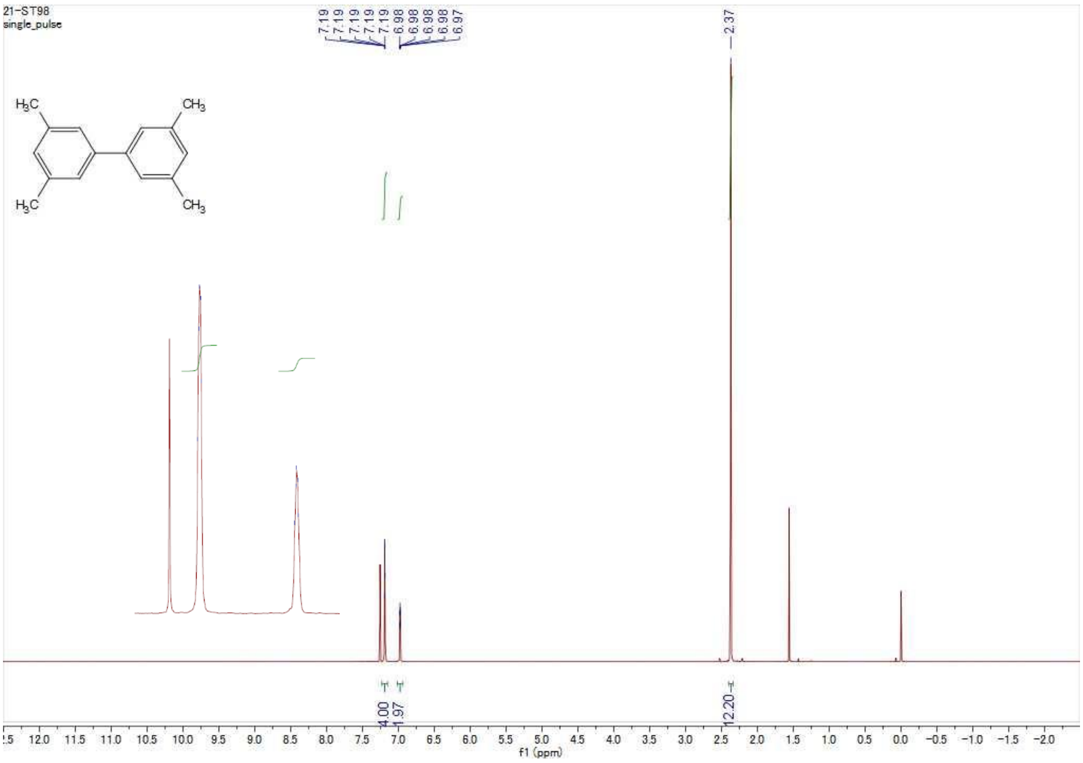

^{13}C NMR of 3,3',5,5'-tetramethyl-1,1'-biphenyl (**3o**)

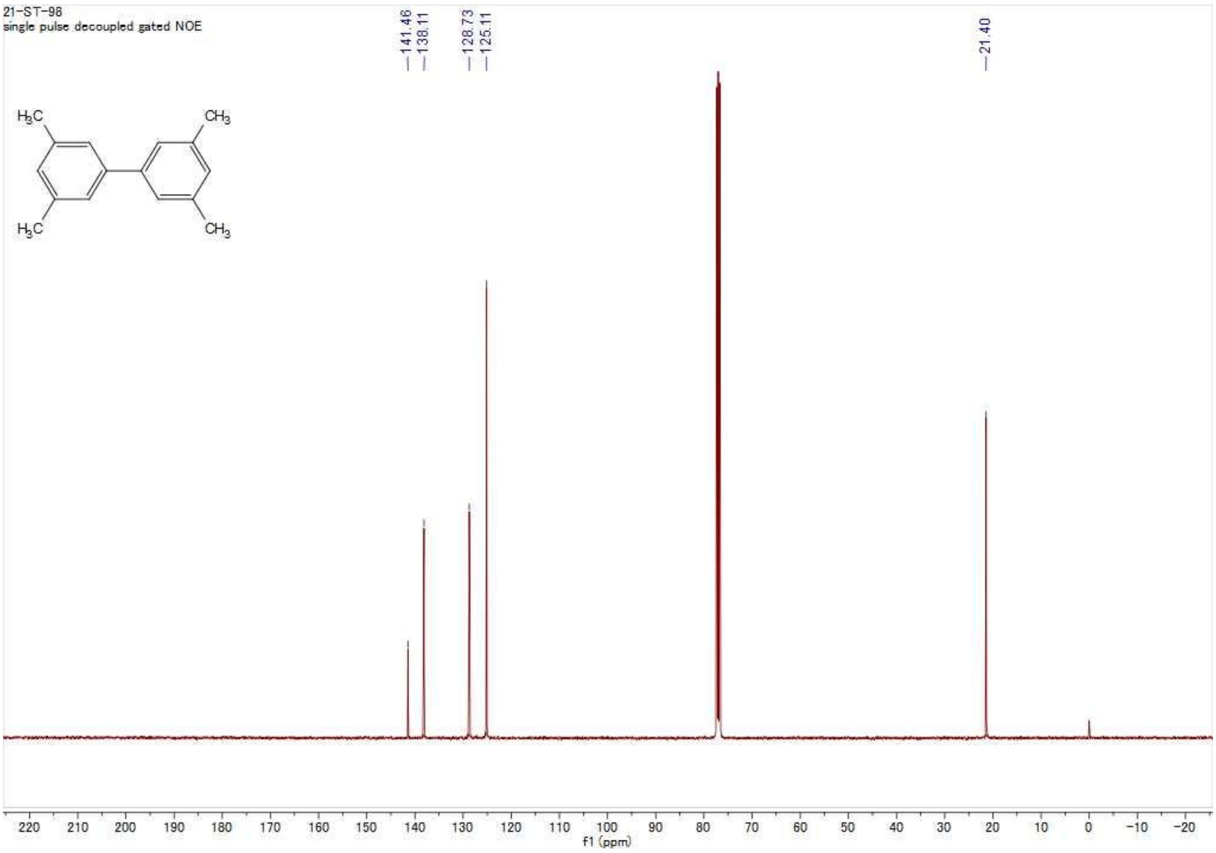

^{1}H NMR of 3,3"-difluoro-1,1':3',1"-terphenyl (**6h**)

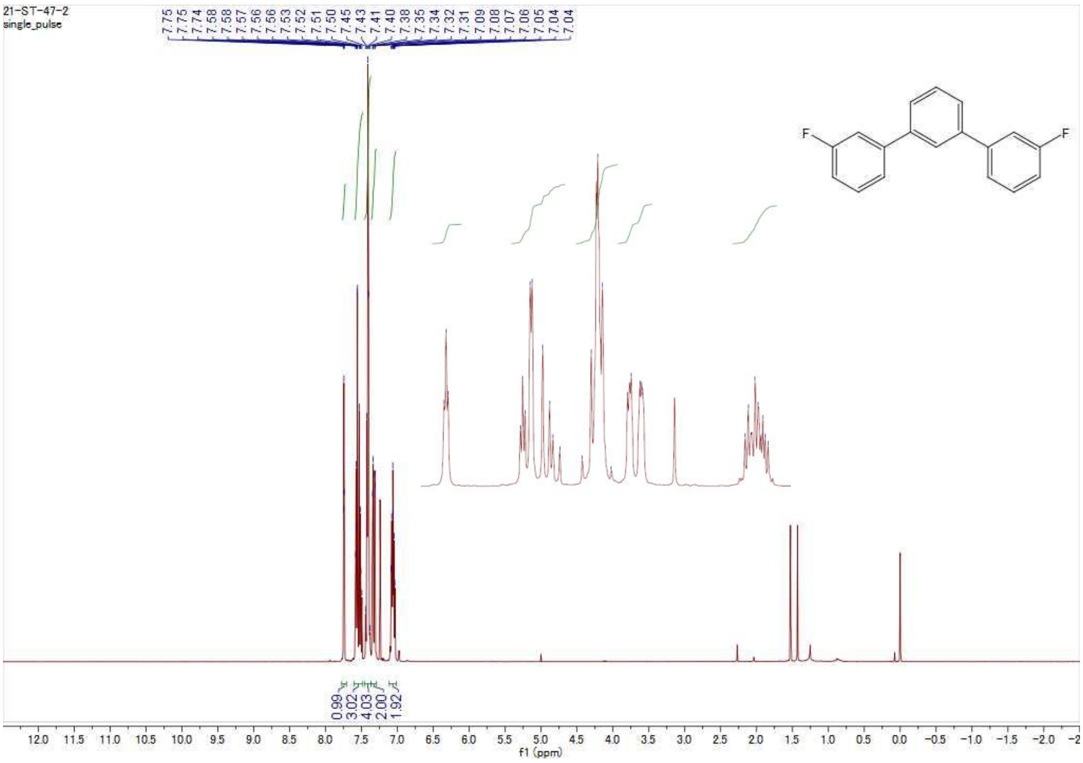

^{13}C NMR of 3,3"-difluoro-1,1':3',1"-terphenyl (**6h**)

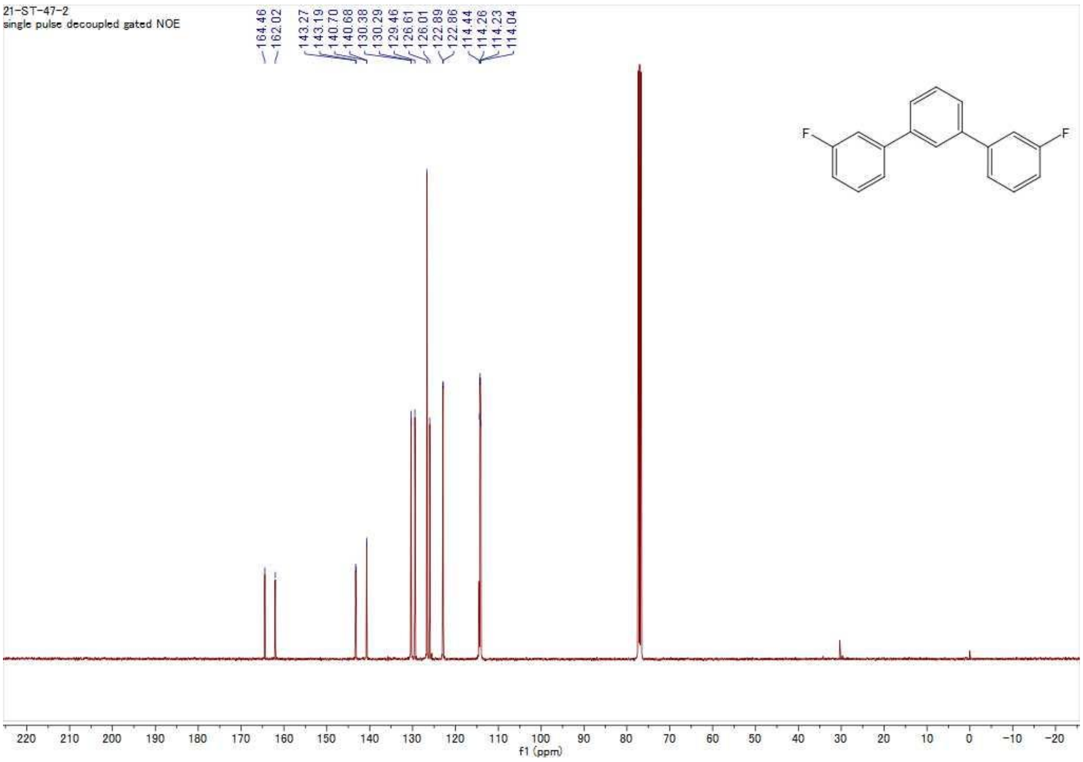

^{19}F NMR of 3,3"-difluoro-1,1':3',1"-terphenyl (**6h**)

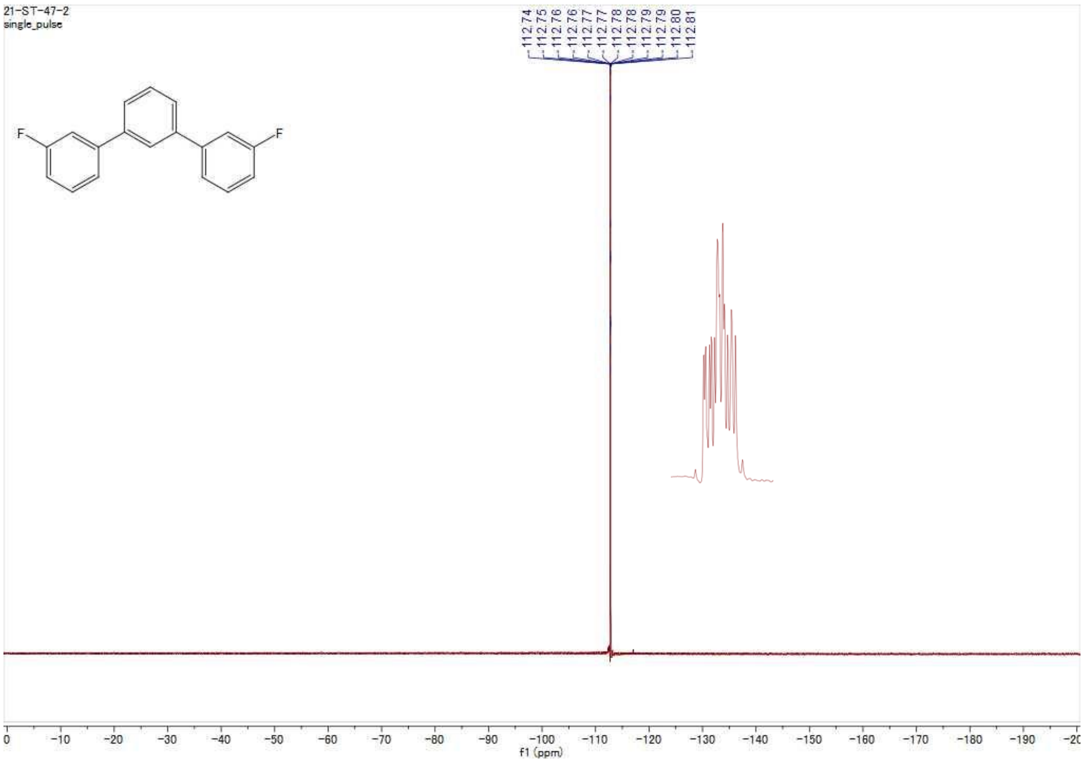

^{1}H NMR of [1,1'-biphenyl]-3,3',4,4'-tetracarboxylic acid (**10n**)

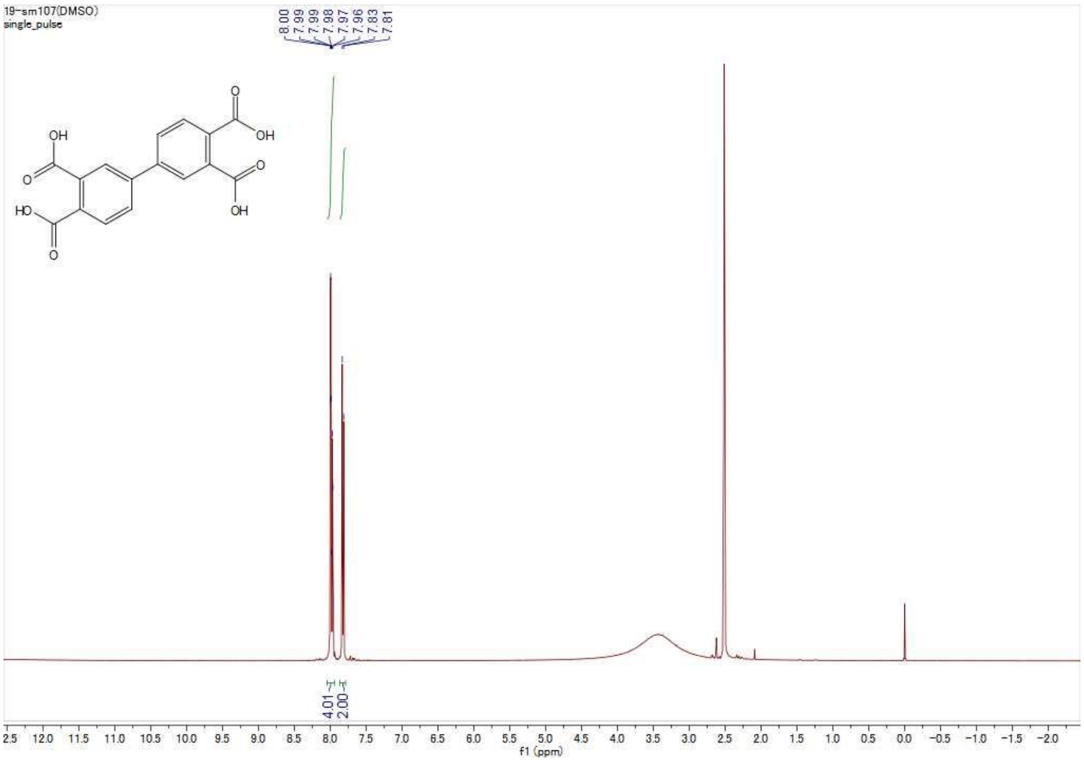

^{13}C NMR of [1,1'-biphenyl]-3,3',4,4'-tetracarboxylic acid (**10n**)

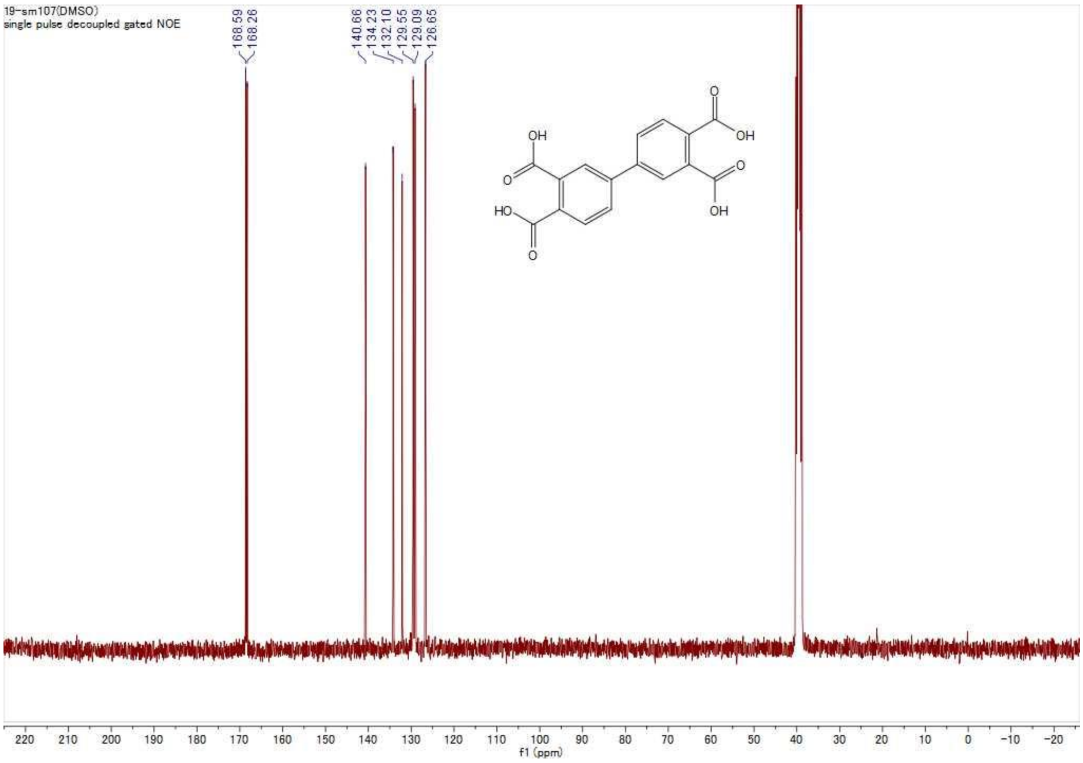

Supplement: File 1 — General procedures and analytical data, including copies of 1H NMR and 13C NMR spectra. [file Beilstein_J_Org_Chem-20-1341-s001.pdf]
